# Alleviation of chronic neuropathic pain by environmental enrichment in mice well after the establishment of chronic pain

**DOI:** 10.1186/1744-9081-9-22

**Published:** 2013-06-07

**Authors:** Pascal Vachon, Magali Millecamps, Lucie Low, Scott J Thompsosn, Floriane Pailleux, Francis Beaudry, Catherine M Bushnell, Laura S Stone

**Affiliations:** 1 Department of Veterinary Biomedicine, University of Montreal, Faculty of Veterinary Medicine, St-Hyacinthe, Quebec, Canada; 2 Alan Edwards Centre for Research on Pain, McGill University, Montreal, Quebec, Canada; 3 Faculty of Dentistry, McGill University, Montreal, Quebec, Canada; 4 Department of Anesthesiology, McGill University, Montreal, Quebec, Canada; 5 Department of Pharmacology & Therapeutics, McGill University, Montreal, Quebec, Canada; 6 Neurology & Neurosurgery, McGill University, Montreal, Quebec, Canada

**Keywords:** Chronic neuropathic pain, Environmental enrichment, Environmental impoverishment, Animal model, Spared nerve injury

## Abstract

**Background:**

In animal models, the impact of social and environmental manipulations on chronic pain have been investigated in short term studies where enrichment was implemented prior to or concurrently with the injury. The focus of this study was to evaluate the impact of environmental enrichment or impoverishment in mice three months after induction of chronic neuropathic pain.

**Methods:**

Thirty-four CD-1 seven to eight week-old male mice were used. Mice underwent surgery on the left leg under isoflurane anesthesia to induce the spared nerve injury model of neuropathic pain or sham condition. Mice were then randomly assigned to one of four groups: nerve injury with enriched environment (n = 9), nerve injury with impoverished environment (n = 8), sham surgery with enriched environment (n = 9), or sham surgery with impoverished environment (n = 8). The effects of environmental manipulations on mechanical (von Frey filaments) heat (hot plate) and cold (acetone test) cutaneous hypersensitivities, motor impairment (Rotarod), spontaneous exploratory behavior (open field test), anxiety-like behavior (elevated plus maze) and depression-like phenotype (tail suspension test) were assessed in neuropathic and control mice 1 and 2 months post-environmental change. Finally, the effect of the environment on spinal expression of the pro-nociceptive neuropeptides substance P and CGRP form the lumbar spinal cord collected at the end of the study was evaluated by tandem liquid chromatography mass spectrometry.

**Results:**

Environmental enrichment attenuated nerve injury-induced hypersensitivity to mechanical and cold stimuli. In contrast, an impoverished environment exacerbated mechanical hypersensitivity. No antidepressant effects of enrichment were observed in animals with chronic neuropathic pain. Finally, environmental enrichment resulted lower SP and CGRP concentrations in neuropathic animals compared to impoverishment. These effects were all observed in animals that had been neuropathic for several months prior to intervention.

**Conclusions:**

These results suggest that environmental factors could play an important role in the rehabilitation of chronic pain patients well after the establishment of chronic pain. Enrichment is a potentially inexpensive, safe and easily implemented non-pharmacological intervention for the treatment of chronic pain.

## Background

The biopsychosocial model suggests that biological, psychological, and social factors all play a role in disease and illness. Providing animals with an enriched environment (i.e. larger housing units, increased access to physical activity, enhanced social interactions), is neuroprotective during aging and in a variety of neurodegenerative disorders, including stroke [1], traumatic brain injury [2], epilepsy [3] and Alzheimer’s disease [4].

A growing body of evidence shows that chronic pain shares many characteristics with neurodegenerative diseases, including the development of co-morbid depression and anxiety [5], reduced brain grey matter [6,7] and disruptions of supra-spinal neural activity [8]. Consequently, environmental manipulation may also modulate chronic pain.

It has been previously shown that the quality of the environment can influence the development of injury-related pain and recovery in rodents. For example, rats exposed to environmental enrichment recover faster from local joint inflammation [9-11] and demonstrate less hypersensitivity [12]. Environmental enrichment also improves sensory and motor dysfunction in rats after spinal cord injury [13], reduces analgesic drug self-administration in mice with post-operative pain [14], and increases the analgesic potency of opioidergic drugs [15-17]. Studies investigating the effects of environmental impoverishment on nociceptive thresholds have reported either increased hypersensitivity or no change; impoverishment therefore has unclear effects on pain sensitivity [18-20].

While the studies described above support the beneficial effects of environmental enrichment on pain, fundamental limitations reduce their clinical impact. First, these works were pre-emptive in nature, with enrichment occurring pre-injury or concurrent with injury; they therefore do not address the common clinical scenario of long-term unattended chronic pain. Second, these studies did not consistently evaluate the effect of environmental enrichment on co-morbid conditions such as depression and anxiety-like behaviors, both of which are decreased in healthy mice raised in an enriched environment [21]. Third, the majority were performed in inflammatory models that resolve naturally within a month. The long-term effects of environmental change on chronic pain are therefore unclear. In the current study, the effects of environmental enrichment on both the sensory and affective components of chronic pain were evaluated in a model of long-term chronic pain, with the environmental changes occurring months after the initial injury.

Following nerve injury, neuroplastic changes are known to occur throughout the neuroaxis including neuronal sprouting, immune cell proliferation, altered primary afferent activity, spinal cord and supraspinal hyperexcitability, alterations in cortical plasticity, and pathological changes in the expression levels of neurotransmitter and modulators, including the pro-nociceptive neuropeptides substance P (SP) and calcitonin gene-related peptide (CGRP) [22-26]. SP and CGRP are expressed by a subset of nociceptive sensory neurons, where they contribute to peripheral and central sensitization in chronic pain conditions [27-30]. Expression levels of these neuropeptides in spinal cord are useful biomarkers for neuroplasticity secondary to peripheral injury [31]. SP and CGRP were therefore used to assess the physiological impact of environmental enrichment on spinal plasticity.

The growing use of non-pharmacological interventions such as cognitive behavioral therapy, support groups, meditation, yoga and physical therapy all contain aspects of social and/or physical enrichment. The study of environmental enrichment in pre-clinical models is therefore of high clinical relevance. The present study assessed the impact of environmental manipulation a) in animals with pre-existing chronic neuropathic pain, b) on changes in neuropeptide expression and c) on both the sensory and affective components of chronic pain. We have previously shown that changes in the mechanical and cold sensitivities are related to changes in the DNA methylation of prefrontal cortex and the amygdala [32]. The article focuses on the time line of these behavioral changes using a more extensive battery of tests and shows the modifications in selected brain related peptides in the spinal cord, occurring with environmental modifications.

## Materials and methods

### Animals and environmental manipulations

Thirty-four CD-1 seven to eight week-old male mice (Charles River, St-Constant, QC, Canada) were used for this study. At arrival, they were kept in a standard laboratory animal environment (fresh filtered air, temperature: 21 ± 2°C, humidity: 40-60% and light–dark cycle: 12:12 h). They were housed 5 per cage, in positive pressure individual ventilated polycarbonate cages (Allentown Inc., Allentown, NJ, USA) with nesting material. They received reverse osmosis water via an automatic watering system and a standard laboratory rodent diet *ad libitum* (Charles River Rodent Chow, St-Constant, QC, Canada).

**Figure 1:**
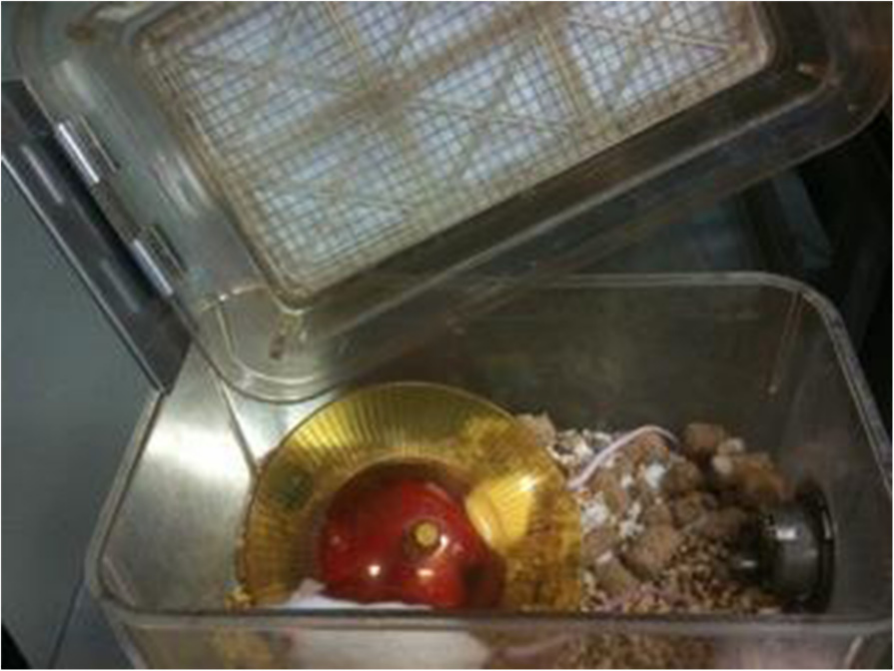
Cage used for environmental enrichment.

Neuropathic pain was induced using the spared nerve injury model (sham surgery as control). Mice were housed in a standard laboratory environment in groups of 5 with nesting material. Three months following injury, behaviors were assessed and mice were randomly assigned to one of four groups: nerve injury with enriched environment (n = 9), nerve injury with impoverished environment (n = 8), sham surgery with enriched environment (n = 9), or sham surgery with impoverished environment (n = 8). The enriched environment included 3 animals per cage that were previously cage mates, a colored plastic hut with a running wheel and marbles (Figure 1). The impoverished environment involved single housing with only cotton nesting materials (i.e. no running wheel, no marbles). The McGill University Institutional Animal Care Committee approved all experiments.

### Spared nerve injury

Following a one-week acclimation period the mice underwent surgery on the left leg under isoflurane anesthesia to induce the spared nerve injury (SNI) model of neuropathic pain or sham condition [33,34]. This model produces persistent long-lasting behavioral signs of neuropathic pain. In brief, the tibial and common peroneal nerves were tied with suture and cut; the sural nerve was left intact. For sham surgery, the nerves were exposed and visualized but not injured. The mice were then returned to their home cage in standard laboratory conditions to allow for the effects of the nerve injury to become chronic for three months.

### Behavioral assays

Mice were tested for nerve injury-induced changes in mechanical sensitivity, temperature sensitivity, depression, anxiety, motor capacity and spontaneous exploratory behavior as described below. Testing occurred at baseline (3 months post-injury but prior to environmental manipulation) and at one and two months following environmental manipulation. All testing occurred between 7 AM and 3 PM.

The mechanical sensitivity was assessed on the plantar surface of both hind paws. Calibrated von Frey Filaments (Stoelting Co., Wood Dale, IL) were applied to the point of bending for 4 sec or until withdrawal. Mice were acclimated to the experimental setup 60 minutes prior to testing. The stimulus intensity ranged from 0.01–4.0 g, corresponding to filament numbers 1.65 – 4.56. The 50% threshold to withdraw was calculated as previously described [35].

The acetone test was used to measure sensitivity to cold. In this assay, a drop (50 μL) of acetone is gently applied to the plantar surface of the hind paw and the total time spent in acetone-evoked behaviors (paw elevation, flinching, licking, and scratching) over a 1-minute observation period is determined.

Mice were placed on an approximately 25 cm^2^ hot plate (IITC Life Science, CA, USA) surrounded by a Plexiglas enclosure to test heat sensitivity. The plate was heated at a constant temperature of 55°C and the latency to response (lick, flick or a hind paw flexion) was measured. A cut off period of 30 sec was set to avoid tissue damage.

The mouse tail suspension assay is used as a model of learned helplessness and is sensitive to antidepressants [36]. Mice were suspended individually underneath a platform by the tail with adhesive tape attached at 0.5 cm from the base of the tail and at 1 cm from the tip of the tail. Behavior was videotaped for 120 seconds. The duration of time spent in immobility was subsequently analyzed by an observer blind to experimental condition.

An elevated plus maze apparatus, built in-house, was placed in a quiet room illuminated with white light to test for anxiety-like behavior. Shaped like a plus sign, it consists of a central platform (5 × 5 cm), two open arms (5 × 30 cm) and two enclosed arms (5 × 30 × 15 cm) opposite to each other. The entire apparatus was elevated to a height of 50 cm above the floor. Mice were individually placed at the intersection of the two arms and their spontaneous behavior was videotaped for 5 minutes and scored by an observer blind to experimental condition. Percentage of the total time spent in the open and closed arms were measured [37].

We used an accelerating rotarod (IITC Life Science, CA, USA) with the mouse adapter (rod diameter = 3.2 cm) to measure motor capacity. The task includes a speed ramp from 0 to 30 rotations per minute over 60 sec followed by an additional 240 sec at the maximal speed. The latency to fall was measured.

Spontaneous exploratory behavior was evaluated with a transparent open field apparatus (24 × 24 cm), placed in a quiet room illuminated with white light. The floor of the apparatus was divided equally into nine squares (8 × 8 cm^2^). Mice were individually placed into the open field on the central square, and their spontaneous behavior was videotaped for 5 minutes before being scored by an observer blind to experimental condition. Subsequent analysis of the central and total number of squares visited was used to assess general motor activity and exploration.

### Peptide quantification

Sodium mono-iodoacetate (MIA) (SigmaUltra, 19148-5G), ethylenediaminetetraacetic acid (EDTA), acetic anhydride 99.5% (Ac2O) and ammonium bicarbonate (NH4HCO3) were obtained from Sigma Aldrich, Inc (Saint-Louis, MO, USA). Substance P and CGRP were purchased from Phoenix Pharmaceutical (Belmont, CA, USA). Acetonitrile was purchased from Fisher Scientific (NJ, USA) and trifluoroacetic acid (TFA), formic acid (FA) and ammonium hydroxide (NH4OH) 28.0-30.0% were purchased from J.T. Baker (Phillipsburg, NJ, USA).

Acetylated neuropeptides were used as internal standards. Ac2O reacts principally with the N-terminal primary amine but also with the lysine primary amine. The reaction was performed as previously described [38]. Briefly, the targeted peptides were diluted in a 0.2 M ammonium bicarbonate buffer (pH 7.5). Two hundred μL of standard peptides solution were mixed with 10 μL of Ac2O (≈ 10,000 molar excess) in a microcentrifuge vial. Ten μL of NH4OH was added and the reaction was stopped after 30 min by further diluting the peptides with a 0.25% TFA solution to obtain a final concentration of 500 pg/μL. The internal standard mixture was tested by HPLC-MS/MS and less than 1% of the original peptides were observed.

Mouse spinal cord tissues were weighed and homogenized using a Tissue-Tearor homogenizer following the addition of 0.25% TFA solution at a ratio of 1:5 (w/v) [39]. The samples were sonicated for 20 min and 150 μL of the homogenate was mixed with 150 μL of acetonitrile to precipitate high molecular weight proteins. The samples were vortexed and centrifuged at 12,000 g for 10 min and 150 μL of the supernatant was transferred into an injection vial then spiked with 150 μL of the internal standard solution. Vials were capped and vigorously vortexed prior to analysis.

The HPLC-MS/MS system is comprised of a Thermo Surveyor autosampler, a Thermo Surveyor MS pump and a Thermo LCQ Advantage Ion Trap Mass Spectrometer (San Jose, CA, USA). Data were acquired and analyzed with Xcalibur 1.4 (San Jose, CA, USA), and regression analyses were performed with PRISM (version 5.0d) GraphPad software (La Jolla, CA, USA) using the nonlinear curve-fitting module with an estimation of the goodness of fit. The calibration lines were constructed from the peak-area ratios of targeted neuropeptides and the acetylated neuropeptide analogue internal standards.

The chromatography was achieved using a gradient mobile phase along with a microbore column Thermo Biobasic C8 100 × 1 mm with a particle size of 5 μm. The initial mobile phase condition consisted of acetonitrile and water (both fortified with 0.4% of formic acid) at a ratio of 5:95, respectively. From 0 to 1 min, the ratio was maintained at 5:95. From 1 to 12 min a linear gradient was applied up to a ratio of 60:40 and maintained for 5 min. The mobile phase composition ratio was reverted to the initial conditions and the column was allowed to re-equilibrate for 15 min for a total run time of 32 min. The flow rate was fixed at 75 μL/min. All targeted neuropeptides and acetylated neuropeptides eluted between 9.8 to 12.2 min. Two μL of sample were injected using full loop mode. The mass spectrometer was coupled with the HPLC system using a pneumatically assisted electrospray ion source (ESI). The sheath gas was set to 10 units and the ESI electrode was set to 4000 V in positive mode. The capillary temperature was set at 300°C and the capillary voltage to 34 V. All scan events were acquired with a 300 ms maximum injection time and the isolation width used for the precursor ions was 3 Da. The mass spectrometer operated for quantitative analysis in full scan MS/MS and quantitation was based on post processing MRM extracted ion chromatograms.

### Statistics

Statistical analyses and graphs were performed using GraphPad Prism 4.0. The time course for sensory, motor and affective measures were analyzed by 2-way repeated measures ANOVA followed by the Bonferroni post-hoc test for multiple comparisons. All data are expressed and plotted as mean ± SEM. P < .01 was considered statistically significant. If a significant effect of environment was observed in the absence of a time effect or an environment x time interaction, 1-way repeated measures ANOVA was used to determine if a time effect existed within each group (i.e. neuropathic enriched). The substance P and CGRP quantifications were analysed with a 2-way ANOVA. When an environmental effect was detected, but the interaction between environment x injury (SNI or Sham) was not significant, the values for enriched versus impoverished were compared by 1-tailed unpaired t-test for neuropathic and sham animals.

## Results

### Impact of environmental manipulation on behavioral signs of chronic neuropathic pain

**Figure 2:**
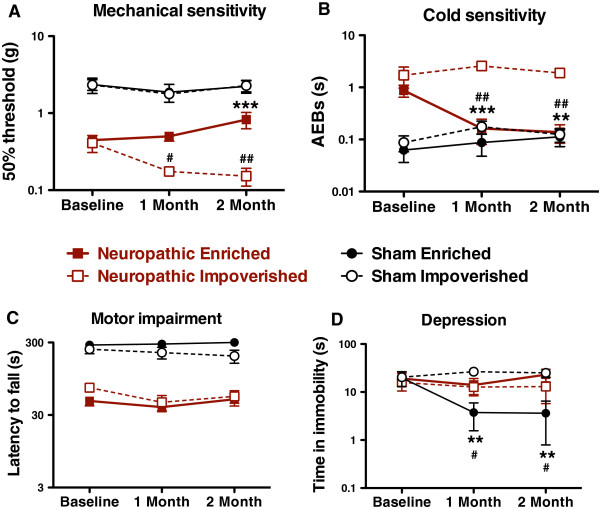
**Effect of environmental manipulation in neuropathic and sham control mice. **Nerve injury-induced hypersensitivity to mechanical (**A**) and cold (**B**) stimuli was attenuated following environmental enrichment and exacerbated by environmental impoverishment (mechanical only). (**C**) The motor impairment observed following nerve injury was insensitive to environmental change. (**D**) Environmental enrichment had significant antidepressant effects in uninjured but not in neuropathic mice. Impoverishment had no effect on this assay in either group. ** p < 0.01, *** p < 0.001, 2-way repeated measures ANOVA followed by the Bonferronni post-hoc test, enriched vs. impoverished within pathological condition (neuropathic vs. sham); # p < 0.05, ## p < 0.05, 1-way ANOVA followed by the Dunnett post-hoc test, effect of time within pathological condition; mean ± SEM, n = 8-9/group.

In neuropathic mice, environmental enrichment significantly reduced hypersensitivity to mechanical (Figure 2A; enriched vs. impoverished, 2-way RM-ANOVA, F_(1, 26)_ = 14.9, p = 0.002) and cold (Figure 2B, enriched vs. impoverished, 2-way RM-ANOVA, F_(1, 26)_ = 37.4, p < 0.0001) stimuli compared to impoverished animals but had no effect on normal heat sensitivity (data not shown, enriched vs. impoverished, 2-way RM-ANOVA, F_(1, 26)_ = 0.11, N.S.; at 2 months enriched = 6.6 ± 0.7 sec, impoverished = 6.8 ± 0.6 sec). Decreased hypersensitivity to cold stimuli was detected compared to pre-enrichment values (Figure 2B, neuropathic enriched vs. time, 1-way RM-ANOVA, F_(2,7)_ = 7.75, p = 0.005). In comparison to pre-impoverished values, the impoverished environment resulted in increased hypersensitivity to mechanical stimuli (Figure 1A, neuropathic impoverished vs. time, 1-way RM-ANOVA, F_(2,7)_ = 6.78, p = 0.009). No significant changes were observed in sensitivity to mechanical, cold or heat in sham control mice.

The motor impairment observed in chronic neuropathic mice was not affected by 2 months of environmental enrichment (Figure 2C; enriched vs. impoverished, 2-way RM-ANOVA, F_(1, 26)_ = 1.31, N.S.). Spontaneous exploratory behavior was also unaltered (data not shown; enriched vs. impoverished, 2-way RM-ANOVA, F_(1, 26)_ = 0.06, N.S.; at 2 months enriched = 71 ± 16 squares traversed, impoverished = 80 ± 8 squares traversed). No significant changes were observed in sham control mice.

In neuropathic mice, neither anxiety (data not shown; enriched vs. impoverished, 2-way RM-ANOVA, F_(1, 26)_ = 3.71, N.S.; at 2 months enriched = 5.6 ± 1.6% time in open arms, impoverished = 5.9 ± 3.2% time in open arms) nor depression-like phenotype (Figure 2D, enriched vs. impoverished, 2-way RM-ANOVA, F_(1, 24)_ = 0.43, N.S.) were affected by 2 months of environmental manipulation. When comparing sham to neuropathic animals, time spent on the open arm progressively decreased for all groups, in a similar fashion, and this was interpreted as a learning process. In contrast, healthy sham control mice subjected to an enriched environment spent significantly less time in immobility in the tail-suspension assay (Figure 2D, sham enriched vs. time, 1-way RM-ANOVA, F_(2, 7)_ = 5.12, p = 0.02), consistent with the previously reported antidepressant effects of environmental enrichment in normal mice [18].

### Impact of environmental manipulation on expression levels of spinal neuropeptides

**Figure 3:**
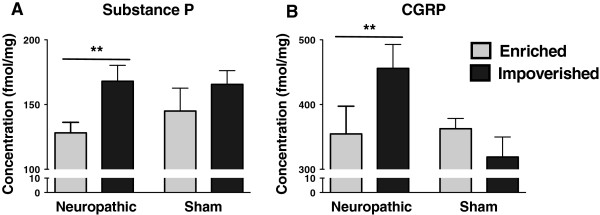
**Effect of environmental manipulation on spinal SP and CGRP content in neuropathic and sham control mice. **Substance P (**A**) and CGRP (**B**) concentrations were lower in the enriched vs. impoverished neuropathic animals, but no significant difference in peptides concentrations was seen in control mice. Since the 2-way ANOVA revealed an environmental effect but no interaction between environment (enriched vs. impoverished) x time, the data was analyzed by unpaired t-test, enriched vs. impoverished, within pathological condition (neuropathic vs. sham). * p < 0.05, ** p < 0.01, mean ± SEM, n = 7/group.

Environmental manipulation resulted in significant changes in the expression of both SP and CGRP. Statistical analyses revealed an effect of environment (2-way ANOVA, F_(1, 24)_ = 5.77, p = 0.026 for SP, F_(1, 24)_ = 3.675, p = 0.068 for CGRP), but no effect of surgery and no interaction between these two factors. In neuropathic mice, both SP and CGRP were significantly less abundant in animals in enriched environment (Figure 3A, B). In contrast, levels of both SP and CGRP were similar in sham animals housed in either enriched or impoverished environments. These data suggest that pain-related peptides play an important role in the recovery of sensory hypersensitivity by environmental change.

## Discussion

### Environmental manipulation and sensory components of chronic neuropathic pain

Three months following spared nerve injury, mice exhibited chronic mechanical and cold hypersensitivity that was associated with motor impairment and signs. Environmental enrichment initiated after the establishment of neuropathic pain alleviated whereas environmental impoverishment exacerbated the condition. The changes in sensitivity are unlikely to reflect non-specific changes in reactivity since sensitivity to heat was unaltered by either nerve injury or environmental manipulation. In contrast, environmental manipulation had no effect on motor impairment or on the affective component of chronic neuropathic pain, but enrichment did have anti-depressant effects in sham control mice. Therefore environmental enrichment did not appear to have any effect on neuropathic animals regarding anxiety-like and depression-like phenotype behaviors.

Environmental enrichment includes both social and physical (i.e. increased activity, exercise) components. In a study comparing these two components on inflammatory pain in rodent, Gabriel et al. demonstrated that physical enrichment has a larger effect than social enrichment on reducing inflammatory pain [11]. The beneficial effects of physical activity are further supported by studies showing that the development of neuropathic pain after sciatic nerve compression is attenuated in rats that received controlled treadmill running [39], or swimming exercise [40].

Clinical studies further suggest that exercise decreases pain symptoms in chronic pain patients. While the intensity of physical activity varies between studies, increased activity improves pain ratings in a broad variety of painful clinical conditions including low back pain, osteoarthritis, and fibromyalgia [41-46].

### Environmental manipulation and affective components of chronic neuropathic pain

Depression and anxiety-like behaviors are highly co-morbid with chronic pain in humans. In the present study, 3 months of continuous neuropathic pain didn’t result in significant signs of anxiety-like behaviors in the elevated plus maze assay. Others have reported the development of anxiety-like behavior in mice and rats after nerve injury [47,48] but these conditions occurred after longer periods of time. In healthy mice, environmental enrichment has been shown to decrease anxiety-like behavior [21], while social isolation significantly increases anxiety-like behavior [49]. In contrast, neither environmental enrichment nor impoverishment significantly altered anxiety-like behavior in sham or neuropathic mice in the current study. Because both the behavioral assay used (elevated maze) and the environmental manipulations (activity wheel or social isolation) were similar to those used in previous studies, the reasons for this difference are unclear.

In rodents, depression-like phenotype is typically assessed using the forced swim test or the tail suspension assay. In both assays, learned helplessness is determined by measuring the time spent in immobility in response to an inescapable stressful situation. Previous studies using this paradigm to assess depression-like phenotype in animals with chronic neuropathic pain have resulted in contradictory conclusions [50-52]. In the current study, neuropathic mice did not demonstrate significant signs of depression-like phenotype in the tail suspension assay. However, consistent with the literature [21], environmental enrichment did have anti-depressant effects in uninjured mice. The lack of similar benefits in nerve-injured mice suggests that ongoing chronic pain generates resistance to the beneficial effects of environmental enrichment on mood.

This study does not provide information on the ongoing nociceptive parameters in animals remaining in the initial housing situation. Although there are clear differences between the impoverished and enriched environmental conditions, a clear conclusion of the effects of the impoverished condition cannot be drawn at the present time.

### Mechanisms underlying environmental enrichment-induced behavioral and neuroplasticity

Environment-mediated neuroplasticity has been reported throughout the CNS [53]. It is therefore likely that the beneficial effects on chronic pain are mediated at both supraspinal and spinal levels.

At the spinal level, environmental enrichment and exercise increase expression of brain-derived neurotrophic factor (BDNF), glial-derived neurotrophic factor (GDNF) and mTOR [54,55] and modulate SP and CGRP levels [56,57]. Supraspinally, exercise modulates the expression of μ-opioid receptors [58], and induces cortical organization of sensorimotor cortex after spinal transection [59]. Environmental enrichment also increases endogenous opioid peptides and BDNF in cerebral spinal fluid and serum [39,60]. In the present study, the modulation of substance P and CGRP with environmental enrichment suggest that spinal cord mechanism are related to nerve injury-induced hypersensitivity to mechanical and cold stimuli. These modifications could be related to supraspinal changes such as prefrontal cortex peptide synthesis modifications that would affect subcortical structures, such as periaqueductal gray and rostroventral medulla, that could modify pain gating in the spinal cord.

A few molecular mechanisms have been proposed to explain the effects of environmental enrichment on pain perception. Elevated levels of BDNF in the central nervous system following enrichment or exercise are neuroprotective and stimulate neurogenesis [61-63] and BDNF modulates brain and spinal cord levels of neuropeptides involved in the regulation of nociception including endogenous opioid peptides and SP [64,65]. Consistent with a role for BDNF-induced neuroplasticity in mediating the antinociceptive effects of exercise, we show that the concentration of SP and CGRP is significantly lower in animals with neuropathic pain in the enriched vs. impoverished environment. However comparing neuropathic and sham results, it is somewhat confusing that in neuropathic animals substance P concentrations was lower with enrichment and that CGRP concentrations were higher with the impoverished environment. Although both peptide concentrations decrease in neuropathic animals with enrichment, we have no clear explanation for this result at the present time. To evaluate the importance of these changes, peptides concentrations should be measured when neuropathy is present (at different time points) and following the environmental modifications. Without baseline data it is difficult the present time to evaluate the importance of these changes in our experiment. Since pain peptide receptors, such as NK1, increase with chronic pain [66], neuropathic animals might be more sensitive to higher concentrations, and this would correlate well with pain behaviors, especially for substance P in neuropathic animals.

Increases in neurogenesis could also play a role in exercise- and environmental enrichment-induced attenuation of neuropathic pain. There are many studies on chronic pain reporting anatomical and functional changes in the prefrontal cortex (PFC), an area also implicated in the common co-morbidities of depression-like phenotype and anxiety-like behaviors in both human [67] and animal studies [68]. We reported using brain imaging methods that pathological changes in both the anatomy and function of the PFC in humans with chronic low back pain can be reversed with effective pain management [7]. The resultant increases in PFC thickness correlated with the reduction of both pain and physical disability. In a recent clinical study, mild exercise in aging adults was associated with elevated serum BDNF, improved cognitive performance and increases in local gray matter volume in the PFC [69]. Thus, the effects of environmental enrichment on endogenous opioids, neuropeptides, neurotrophic factors and the prefrontal cortex may all be involved in the resolution of chronic pain.

## Conclusion

Individuals with chronic pain may suffer for years without receiving relief. Although the translation from rodent studies to the clinical situation is sometimes difficult, pre-clinical experiments may lead to a better understanding of the effects of interventions on pre-existing chronic conditions. In this study, we tested the therapeutic effects of environmental enrichment several months following the induction of neuropathic pain. The ability to reverse well-established chronic pain with lifestyle changes has important therapeutic implications. Elucidation of the molecular mechanisms contributing to these positive effects will lead to novel pharmacological and non-pharmacological treatment strategies, ultimately improving the quality of life of individuals suffering from chronic pain.

## Abbreviations

AEB’s: Acetone-evoked behaviors; Ac2O: Acetic anhydride; ANOVA: Analysis of variance; BDNF: Brain-derived neurotrophic factor; CGRP: Calcitonin gene related peptide; EDTA: Ethylenediaminetetraacetic acid; ESI: Electrospray ion source; FA: Formic acid; GDNF: Glial-derived neurotrophic factor; HPLC-MS/MS: Tandem liquid chromatography mass spectrometry; mTOR: Mammalian target of rapamycin; NH4OH: Ammonium hydroxide; NH4HCO3: Ammonium bicarbonate; PFC: Prefrontal cortex; RM: Repeated measures; SEM: Standard error of the mean; SP: Substance P; SNI: Spared nerve injury; TFA: Trifluoroacetic acid.

## Competing interests

The authors have no competing interests to declare.

## Authors’ contributions

PV – Study design, animal testing, tissue preparation, manuscript preparation. MM - Study design, SNI and sham surgery, tissue preparation, statistical analysis, figure & manuscript preparation. LAL – Statistical analysis, figure & manuscript preparation. SJT - Tissue preparation, manuscript preparation. FP - Tissue processing and peptide quantification. FB - Tissue processing and peptide quantification. MCB - Study design, manuscript preparation. LSS - Study design, statistical analysis, figure & manuscript preparation. All authors read and approved the final manuscript.
